# Entropy and the Limits to Growth

**DOI:** 10.3390/e26060489

**Published:** 2024-05-31

**Authors:** Reiner Kümmel

**Affiliations:** Institute for Theoretical Physics und Astrophysics, University of Würzburg, D-97074 Würzburg, Germany; reiner.kuemmel@uni-wuerzburg.de

**Keywords:** economic growth, capital, labor, energy, entropy, constraints, space manufacturing

## Abstract

In its business-as-usual scenario, the 1972 Club-of-Rome report—The Limits to Growth—describes the collapse of the world economy around the year 2030, either because of the scarcity of natural resources or because of pollution. Mainstream economists, the high priests of secular societies, condemned it fiercely. Their gospel of perpetual economic growth, during which technological progress would solve all problems, promises a bright future for all mankind. On the other hand, engineers, natural scientists, and mathematicians realized that the breakdown scenario is due to the inclusion of the First and the Second Law of Thermodynamics in the Club-of-Rome’s world model. According to these laws, nothing happens in the world without energy conversion and entropy production. In 1865, Rudolph Clausius, the discoverer of entropy, published the laws as the constitution of the universe. Entropy is the physical measure of disorder. Without a proper understanding of energy and entropy in the economy, all efforts to achieve sustainability will fail.

## 1. Introduction: An Old Man Recalls How He Finally Grasped Entropy and the Rest

In 1972, I was shocked by *Time Magazine’s* preview of the research report *The Limits to Growth* (LTG) [1], which was presented to the Club of Rome.

It was my third year in the Physics Department of Universidad del Valle in Cali, Colombia, where I worked within a cooperation between Colombian universities and German institutions of academic development assistance. The mission was to support the build up of a new Masters program in physics, initiated by Colombian professors who before had studied abroad. They realized the need for thorough graduate education in the natural sciences and engineering in order so that their beautiful country with plenty of natural resources has competent people for its belated transition from agrarian feudalism to an industrial society. The wealth of material goods produced by industry’s energy-driven machines would allow raising the standard of living for everyone.

Teaching physics to brilliant students of all skin colors was pure joy, even in times of revolutionary turmoil on campus and military occupation of the university. The students continued taking classes and exams whenever and wherever it was possible. Observing how their brains opened up to the strict, quantitative reasoning of the natural sciences was highly rewarding.

My shock resulted from LTG’s message that progress of global industrialization along the path taken by Europe and the USA would ruin the world within the first half of the 21st century. Thus, my attempt to contribute a bit to the industrialization of Colombia would also contribute to global disaster in the future. I took the warnings of LTG seriously, because for the first time I had really understood *entropy*, thanks to my Colombian colleagues who had obliged me to teach thermodynamics in the Masters program and recommended excellent literature. I gathered that the dire outcome of the computer runs in the *business as usual* (BAU) scenarios is due to the inclusion of the first and the second law of thermodynamics in LTG’s World Model. Since these laws are the most powerful laws of nature, I felt that the clarion call of LTG deserves the highest attention. This was confirmed by a series of interdisciplinary seminars of teaching staff and students at the University of Würzburg on LTG and the fierce international debates this book had stimulated [2]. I participated in them after having joined the university in 1974.

Fifty years later, we worry about climate-changing CO_2_ emissions. By now, nature has proven the Club of Rome’s LTG report to be right. Emissions impose the limits to grow on Earth.

## 2. Quarrels about the Limits to Growth

The following quotations from [2] and other sources highlight the need to incorporate the laws of energy conservation and entropy production in economics.

In 1973, professor of economics Egon Tuchtfeld published a peculiar interim assessment of the discussion on the Limits to Growth [3]. His paper shows that he had clearly seen that LTG’s World Model is based on system dynamics, an offspring of engineering and the natural sciences. He also states that exponential industrial growth in a finite system cannot prevail. Despite that, Tuchtfeldt polemicizes the following: “Dreamers in futurology and money makers endowed with a sixth sense can no longer keep themselves from discharging their phantasies and inundate the book market. ‘Back to the stone-age’ is the apocalyptic vision. A preliminary highlight is undoubtedly …the Report to the Club of Rome. …The smart presentation of the material by numerous figures, graphics, and tables conveys the impression of understandability to the interested layman. No wonder, here was ‘women’s lib’ in action. At the beginning of 1973 …D.L. Meadows revealed the result of the emancipatory development of his wife with typical American airiness: ‘By the way, we owe this popular report on our study essentially to my wife Donella’. …The report also activates another contemporary myth, namely the myth of the team work. …Unfortunately, the report fails to inform about the number of beer crates the team has consumed during its production”. (For the German original text see [2] (p. 95)).

In 2022, a commentary entitled “Ein Schauermärchen” appeared in an influential weekly magazine [4]. It proclaimed the following: The Club of Rome’s report is completely wrong. It is only good for the trash bin. It severely underestimated the global quantity of material natural resources. The collapse scenario because of their scarcity is an unfounded horror tell tale. Technical progress will solve all problems anyway.

These are just two scorchers of LTG—half a century apart. Many more are quoted by Wikipedia [5], which concentrates on discussing resource scarcity as the reason for the collapse about 2030, as does much of the scholarly criticism. However, Wikipedia’s Figure “World3 Model Standard Run as shown in *The Limits to Growth*” is only *one* of LTG’s *three* scenarios of collapse at about the same time. A few pages behind this first scenario, the book [1] shows the collapse for *twice* as many resources as in the Standard Run, and in Chapter IV on “Technology and the Limits to Growth”, the collapse occurs at *unlimited* resources of energy and materials. In these two scenarios, the world breaks down because of *pollution*.

Here, *entropy* enters the stage of the drama we are actually living in.

## 3. Entropy—The Nasty Twin of Energy

An important non-negative comment on LTG quoted by [5] is that of Robert M. Solow. He had been a vocal critic of LTG. But in 2009, he said that “thirty years later, the situation may have changed… it will probably be more important in the future to deal intellectually, quantitatively, as well as practically, with the mutual interdependence of economic growth, natural resource availability, and environmental constraints” [6]. This is most remarkable, because Solow was awarded the 1987 Nobel Prize in Economic Sciences for his important contributions to the theory of economic growth. When his mathematical analyses revealed that the observed growth of gross domestic product (GDP) in industrial countries is much larger than the one computed with the classical production factors of capital and labor, he introduced the concept of *technological progress* to account for the big difference. This difference became known as the “Solow Residual”. (Taunters refer to it as “The Holy Grail of Economics”.) Numerous sophisticated, formal studies have been dedicated to it. Notwithstanding, Solow stated in 1994 that the dominating role of technological progress “has led to a criticism of the neoclassical model: it is a theory of growth that leaves the main factor in economic growth unexplained” [7].

Since the mid 1970s, there have been publications that added the factor *energy E* to the production factors *capital K* and *labor L* in the theory of economic growth; see, e.g., [8]. This is performed in two ways, which differ in the economic weights (output elasticities) assigned to *K*, *L*, *E*; these are defined in Section 4, Equation (24), for two different sets of constraints. Mainstream economists believe that the economy operates in an equilibrium where the cost–share theorem is valid. This theorem results from the optimization of profit or overall welfare (time-integrated utility). According to this theorem, the economic weights of *K*, *L*, *E* are equal to their shares in total factor cost. In industrial countries, these shares are roughly 25% for capital *K*, 70% for labor *L*, and a meager 5% for *E*. Including energy in this way in growth theory had hardly any impact on the Solow Residual. However, the cost–share theorem is invalid at the energy prices we have known so far [8,9]. Determining *econometrically* the economic weights α of capital, β of labor, and γ of energy yields the time averages of labor $\bar{\beta }$ to be much smaller and those of energy $\bar{\gamma }$ to be much larger than the respective cost shares [8,10,11]. The discrepancy between an output computed with α,β,γ, and the observed output is much smaller than the Solow Residual; this is attributed to the contribution of human ideas, inventions, and value decisions to economic growth [8]. Industrial economies operate far from the equilibrium of mainstream neoclassical economics [12], where the factor energy, which *activates* the capital stock, provides a cornucopia of material wealth.

Solow also pioneered research in economic optimization. Optimization of profit, or overall welfare discussed below in Section 4, depends critically on the *constraints* to which the calculus is subjected. The disregard of *technological* constraints in this optimization led to the hitherto invalid cost-share theorem. Solow’s abovementioned need “to deal …with the mutual interdependence of economic growth, natural resource availability, and environmental constraints” indicates that he not only saw the problems with the fuzziness of “technological progress” but also felt that another constraint may actually endanger economic growth. Solow (* 23 August 1924, † 21 December 2023) had perhaps sensed the predicament of our universe, written down by Rudolph Clausius in 1865 as its constitution and named *The First and The Second Law of Thermodynamics*. These laws say the following: *(1) The Energy of the World is Constant. (2) The Entropy of the World Strives Toward a Maximum* [13].

May philosophers and theologians wonder why we cannot have benign, wealth-creating energy conversion without nasty, polluting entropy production. We restrict ourselves to take a closer look at entropy.

### 3.1. Entropy and Accessible States

Statistical physics, presented by F. Reif in excellent combination with phenomenological thermodynamics [14], delivers the key to understanding entropy via the fundamental postulate of equal a priori probabilities: **an isolated system in equilibrium is equally likely to be in any of its accessible states.**

Let us have a semi-serious look into the number Ω of *many-body* states that are accessible to a system of many *single* objects.

**Figure 1:**
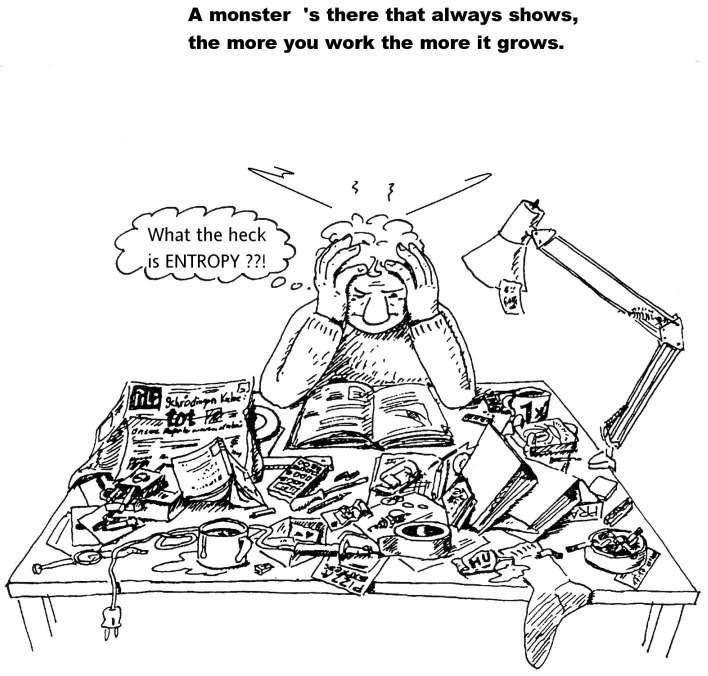
Many natural scientist never become really familiar with entropy (modified cartoon from [15] in [8]).

The cartoon in Figure 1 shows one many-body state on the desk of a physicist who wonders “What… is ENTROPY?”. It consists of many different *single* macroscopic objects. (In quantum mechanics, a many-body state is formed by interacting electrons, protons, neutrons, and other microscopic particles). Obviously, the many-body state in Figure 1 is a mess. Furthermore, there is a big number of similar states that can be obtained from it by just changing the positions of the objects. Moreover, the overwhelming majority of all the resulting possible states also conveys the impression of disorder. Now, let Ω be the number of many-body states for a given quantity of single objects, which are constrained, e.g., in space. The larger this quantity is, the larger Ω will be, and the number of states that look disorderly as well. On the other hand, if Ω is considerably larger than 1, a further increase of it by some small amount δΩ increases messiness by less than that amount: adding another pen or note to the objects on the desk in Figure 1 hardly changes the overall picture. A function that reflects this, because it increases more slowly than Ω, is the natural logarithm ln of Ω. This graphic example makes it perhaps intuitively clear that lnΩ is related to disorder. Multiplying it with the Boltzmann constant $k_{B}=1.38\times 10^{-23}$ J/K, one obtains *Entropy S*, the physical measure of disorder:

(1) $$S\equiv k_{B}\ln\Omega .$$

How statistical physics derives the number Ω of states accessible to a many-body system in equilibrium, and how it arrives at Equation (1), is indicated in [8] (pp. 120–126).

### 3.2. Entropy Production, Emissions, and Limits to Growth

Let us consider a system that has the total volume *V*; *O* is the surface of that volume and defines the system boundaries. (We may think of a northern industrial country and the atmosphere above its land area.) At a given instant of time *t*, the total system entropy is S(t). During an infinitesimal time interval dt, this entropy changes by dS. The change may be due to an exchange $d_{a}S$ of entropy with the environment (this may be the entropy input from solar radiation during the day or the entropy export into space by infrared radiation, especially at night) and to internal entropy production $d_{i}S$ because of irreversible processes (occurring, e.g., in the cars, steam turbines, and blast furnaces of the country):

(2) $$dS=d_{a}S+d_{i}S.$$

In general, real-life processes $d_{a}S$ may be positive or negative (e.g., positive on a cloudy day when a warm wind blows from the south and negative during a clear night, when heat is radiated into space). With Equation (2), the total time change of entropy is

(3) $$\frac{dS}{dt}=\frac{d_{a}S}{dt}+\frac{d_{i}S}{dt}.$$

Whether total entropy S(t) increases, decreases, or stays constant depends upon the magnitudes of $d_{a}S/dt$ and $d_{i}S/dt$, and the sign of $d_{a}S/dt$. The *sign* of $d_{i}S/dt$, however, is known. It is always positive for irreversible processes, i.e., the processes that run in real life:

(4) $$\frac{d_{i}S}{dt}>0.$$

Equation (4) follows from overwhelming empirical evidence and is the most *general* formulation of the Second Law of Thermodynamics. It holds in all systems, whether they are open or closed. (*If* the system boundaries are such that neither energy nor matter can cross them, the system is *closed* and $d_{a}S=0$. Then entropy increases as long as irreversible processes occur. These processes cease when equilibrium is reached and entropy is maximum).

In order to relate entropy production to emissions, which result in pollution and the ultimate limits to growth in the biosphere, we subdivide the total volume *V* of the system in elements ∆V that are small on a macroscopic scale but still so large on an atomic scale that they contain a huge number of molecules with *K* different masses $m_{k}$; $\rho_{k}$ is the mass density of molecules of type *k*, and ρ is the total mass density. As long as temperature variations along an edge of the box are small compared to the average temperature in the box, one has local thermodynamic equilibrium. The same is true for transport processes in gases, if the variation of temperature along the mean free path—i.e., the average distance a gas atom travels between two collisions—is small compared to the temperature itself; further, for compression and expansion processes, the time during which a macroscopically noticeable change in volume by, say, 1 percent occurs, must be much larger than the relaxation time within which internal equilibrium between the atoms is reestablished after a perturbation. Most irreversible processes associated with human activities can be described with the assumption of *local* thermodynamic equilibrium and locally defined thermodynamic variables.

The Second Law, Equation (4), can be cast in a form that shows how it rules emissions. For this purpose, we define s(r,t) as mass-specific entropy; thus, ρs(r,t) is entropy density and $S=\int_{V}\rho sdV$. Furthermore, $J_{S}\left(r,t\right)$ is entropy current density and $\sigma_{S}\left(r,t\right)$ is entropy production density.

By definition, we have

$$\begin{array}{ccc} \frac{dS}{dt} & = & \int_{V}\frac{\partial \rho s}{\partial t}dV,\\ \frac{d_{a}S}{dt} & = & -\int_{O}J_{S}\left(r,t\right)dO\\ & = & -\int_{V}\nabla J_{S}dV,\\ \frac{d_{i}S}{dt} & = & \int_{V}\sigma_{S}\left(r,t\right)dV. \end{array}$$

Here,

$$\nabla J_{S}\equiv \partial J_{Sx}/\partial x+\partial J_{Sy}/\partial y+\partial J_{Sz}/\partial z\;\left(\equiv \mathrm{div}J_{S}\right).$$

The combination of these definitions with Equation (3) results in the entropy balance equation

(5) $$\int_{V}\left[\frac{\partial \rho s}{\partial t}+\nabla J_{S}-\sigma_{S}\right]dV=0.$$

Since Equation (5) is true for any arbitrary volume *V*, the integrand itself must vanish, and we obtain the *local* entropy balance equation

(6) $$\frac{\partial \rho s}{\partial t}+\nabla J_{S}=\sigma_{S}\left(r,t\right).$$

The all-important information added to this equation by the Second Law is that the *density* of entropy production is always *positive* for irreversible processes:

(7) $$\sigma_{S}\left(r,t\right)>0.$$

In transport processes, where there is a small system volume ∆V, which is centered around the point **r** at time *t* and has the velocity **v**, one finds that the change of entropy *within* this moving volume is given by the *substantial* entropy balance equation:

(8) $$\rho \frac{ds}{dt}+\nabla j_{S}=\sigma_{S}\left(r,t\right),$$

where

(9) $$j_{S}\equiv J_{S}-s\rho v$$

is the conductive entropy current density.

The combination of Gibb’s fundamental equation in nonequilibrium thermodynamics with the (substantial) balance equations of mass, momentum, energy, internal energy, concentration $\rho_{k}/\rho$, and entropy are indicated in [8] (pp. 154–167); the detailed mathematical manipulations are in [16] (Chapter 14). The resulting entropy production density is

(10) $$\sigma_{S}=\sigma_{S,dis}\equiv j_{Q}\nabla \frac{1}{T}+\sum_{k=1}^{K}j_{k}\left(-\nabla \frac{\mu_{k}}{T}+\frac{f_{k}}{T}\right)>0.$$

(If chemical reactions occur, one also has chemical entropy production density $\sigma_{S,chem}$. This consists of scalar currents and forces. The dissipative entropy production density $\sigma_{S,dis}$ consists of vectorial currents and forces. Both must be larger than 0 separately in order for $\sigma_{S}>0$).

The spatial derivatives in the gradient operator ∇ operate on the absolute temperature *T* and the chemical potentials $\mu_{k}$ of the particles of type *k*. The gradients of temperature and chemical potentials, and specific external forces $f_{k}$, which act on the particles, represent the generalized forces that drive the heat current density $j_{Q}$ and the diffusion current densities $j_{k}$. In each point, all diffusion current densities cancel so that total mass is conserved everywhere: $\sum_{k=1}^{K}j_{k}=0.$

Equation (10), i.e., the Second Law of Thermodynamics for systems in local thermodynamic equilibrium says the following: In irreversible processes, heat currents of density $j_{Q}$ carry away degraded energy, and diffusion currents of densities $j_{k}$ spread matter in space. The latter, the dissipation of matter, has been emphasized by *Nicholas Georgescu-Roegen* (GR). Calling this the “fourth law of thermodynamics” [17,18] stimulated controversial and lively discussions—see, for example, [19]. These ended with the consensus that the Second Law takes care of the dissipation of energy *and* matter and that GR’s seminal book “The Entropy Law and the Economic Process” [20] awakened society to the relevance of thermodynamics for its future.

Equation (10) is important from an ecological point of view. It tells us that entropy production is unavoidable (>0), whenever inhomogeneities (gradients) and forces “make something happen”, and that entropy production generates emissions of heat and matter in every point of the system. The currents due to $j_{Q}$ and $j_{k}$ carry out the mandate of the Second Law to distribute energy and matter as evenly as possible in space and over the states of motion.

Heat currents that dump waste heat into the environment increase energy’s useless component, *anergy*, and reduce the useful component, *exergy* (with *x*), in the law of energy conservation:

(11) Energy=Exergy+Anergy.

In the nonequilibrium system of industrialized planet Earth, heat and particle currents emanate from furnaces, reactors, and heat engines. These emissions change the energy flows through and the chemical composition of the biosphere to which the living species and their populations have adapted in the course of evolution. If these changes are so big that they cannot be balanced by the biological and anorganic processes driven by the exergy input from the Sun and the radiation of heat into space, and if they are so rapid that biological, social, and technological adaptation deficits develop, the emissions are perceived as environmental pollution.

Pollution by molecules like sulfur dioxide (SO_2_), nitrous oxide (NO_X_, computed on the basis of NO_2_), carbon dioxide (CO_2_), etc. can be converted to thermal pollution by appropriate technologies of NO_X_ abatement, desulfuration, carbon capture and storage (CCS), etc. and the energy required for their operation. Presently, the resulting, inevitable heat emissions do not bother much. But this would change if the world energy consumption per year was to increase by a factor of about 20 over the consumption in the first two decades of the 21st century. (The 2004 level was $1.34\times 10^{13}$ watts (W). Since then, global primary energy consumption has increased by roughly a factor 1.3. In the year 2022, somewhat less than 15,000 million tons of oil equivalents (MtOE) were used [21]). Since most of the consumed energy ends up as heat, total heat emissions would then approach the *heat barrier* of about $3\times 10^{14}$ W. This is roughly 0.2% of the power the Earth receives from the Sun. Anthropogenic heat flows of that magnitude are likely to cause climate changes even without the anthropogenic green house effect [8] (p. 149), [22]. In this sense, the heat barrier is the ultimate thermodynamic limit to growth in the biosphere.

Entropy production (10) not only causes ecological problems but also constrains the industrial generation of wealth. All the machines of capital stock *K* are driven by the component *exergy* of the production factor energy *E*. Their operation dissipates energy via the heat current densities $j_{Q}$. This increases anergy and decreases exergy in Equation (11). Thus, even if sophisticated heat-recovery technologies like heat-exchanger networks, heat pumps, and cogeneration of electricity and heat are implemented, a fixed initial quantity of (primary) energy (measured, e.g., by tOE) becomes completely useless in the end, so the industrial usefulness of “natural resource energy” is definitely limited. Solow may have sensed this threat from the entropy law, Equation (10), when he said the following in 1974: “The world can, in effect, get along without natural resources …(But) if output per unit of resources is effectively bounded—cannot exceed some upper limit of productivity which in turn is not far from where we are now—then catastrophe is unavoidable” [23].

## 4. Wealth Generation and the Sages of Economics

### 4.1. Optimizing Industrial Production

The production factors of an industrial economy, capital *K*, labor *L*, and energy *E*, were introduced in Section 3. Human ideas, inventions, and value decisions matter in the entrepreneurial selections of K,L,E at time *t*.

The capital stock *K* consists of all energy-converting devices and information processors together with all buildings and installations necessary for their protection and operation. It is manipulated and supervised by people, who constitute the production factor *L*. The machines of the capital stock are activated by energy (more precisely, exergy), which is the production factor *E*. The factors cooperate in the generation of the economic output *Y* via work performance and information processing. If the system considered is the whole economy, *Y* is the gross domestic product (GDP).

In the course of economic evolution and technological progress, the factor combinations, which are chosen by entrepreneurs, change. With the increasing density of transistors on a microchip, the degree of automation of the capital stock, ρ(K,L,E), has grown. Recessions and recoveries in the wake of economic shocks like the oil-price explosions caused by the Yom Kippur war in 1973 and the Iran–Iraq war in 1979–1981, the 2008 collapse of the Lehman Brothers Bank, and Russia’s aggression against Ukraine since 2022, varied the degree of capacity utilization η(K,L,E) of the capital stock, when entrepreneurs reacted to supply-side and/or demand-side changes.

Presently, all countries strive for the growth of gross domestic product Y(K,L,E;t). Most people think that the economic activities measured by the GDP indicate wealth and power. The esteem and success of political leaders usually increase with the growth rates of GDP during their rule. That is why research that takes into account limits to growth—for instance, Niko Paech’s book “Liberation from Excess” [24] with its convincing qualitative economic arguments for changing the way we produce and consume—is held in high esteem by those who worry about environmental problems but less favored by mainstream economists, who prefer mathematical reasoning.

In secular industrial societies, economic sages are the top advisers of governments. Especially, winners of (or candidates for) the Nobel Memorial Prize in Economic Sciences play a role similar to that of high priests in former agrarian societies. The hitherto invalid and misleading cost-theorem, addressed in Section 3, results from the disregard of technological and thermodynamic constraints in the optimization of profit or overall welfare. A prominent user of this theorem is W. Nordhaus, who developed the DICE model for research in climate change [25]. He became one of the two 2018 Nobel Laureates in Economics. In his Nobel Lecture “Climate Change: The Ultimate Challenge for Economics”, the gist of what he writes on Slide 4, “The mathematics of the DICE model” [26], is as follows:

**Maximize** *overall welfareW*, which is given by the time integral of the utility function U[c(t)] discounted by the factor $\rho_{d}$:

(12) $$W\equiv \left[\int_{0}^{\infty }U\left[c\left(t\right)\right]e^{-\rho_{d}t}dt\right],$$

taking into account that maximization is subject to a constraint on *consumption* c(t) at time *t*:

(13) c(t)=M[y(t);⋯];

in general, *M* depends on several variables [y(t);⋯].

The *technological* constraints that kill the cost-share theorem [8,9] are ignored by Nordhaus. A sketch of them follows.

### 4.2. Technological Constraints on K,L, and *E*

Capital, labor, and energy can be treated as independent variables within a certain region of positive K,L,E-space. This region is constrained technologically by the limit to the **degree of capacity utilization η(K,L,E)** and the limit to the **degree of automation ρ(K,L,E)**. As pointed out in [8,9], we have

(14) $$\eta \left(K,L,E\right)=\eta_{0}^{*}{\left(\frac{L}{K}\right)}^{\lambda }{\left(\frac{E}{K}\right)}^{\nu },\;\rho \left(K,L,E\right)=\eta \frac{K}{K_{m}\left(Y\right)}\;.$$

The parameters $\eta_{0}^{*}$, λ, and ν can be determined from empirical data on capacity utilization. $K_{m}\left(Y\right)$ is the capital stock that would be required for maximally automated production of a given output *Y*; in this state of the economy, an additional unit of routine labor would no longer contribute to the growth of output.

Obviously, η(K,L,E) cannot exceed unity, because the capital stock cannot work at more than full capacity. (Energy inputs that exceed the power limits the machines are designed for do not make more productive use of the capital stock. Rather, they may damage the machines; too much heating or cooling of rooms is also counterproductive. Nor does it make sense to employ more workers than a production system, working at full capacity, requires for its operation and maintenance).

Furthermore, in a given state of technology at time *t*, the degree of automation of the capital stock has a limit $\rho_{T}\left(t\right)$. This limit depends on the mass and the volume of the energy-conversion devices and information processors that the production system can accommodate when producing the output *Y*. The outermost limit to automation is 1, when $K=K_{m}\left(Y\right)$ and η=1.

Thus, the technological constraints on the combinations of capital, labor, and energy are

(15) $$\eta \left(K,L,E\right)\leq 1\;,\;\rho \left(K,L,E\right)\leq \rho_{T}\left(t\right)\leq 1\;.$$

The behavioral assumptions that firms maximize profit and individuals maximize welfare originate from microeconomics. They are also applied to macroeconomic systems [27]. Much more important than the difference between the two behavioral assumptions is the difference between neglect and non-neglect of the technological constraints (15) when calculating the equilibria in which macroeconomic systems supposedly operate at a given time *t*. Let us look into the necessary condition for welfare maximization.

For notational convenience, we identify K,L,E with the components $X_{1},X_{2},X_{3}$ of the vector

(16) $$X=\left(X_{1},X_{2},X_{3}\right)\equiv \left(K,L,E\right).$$

In this notation, and with the help of slack variables $X_{\eta }$ and $X_{\rho }$, the constraints (15) can be brought into the form of equations,

(17) $$f_{\eta }\left(X,t\right)=0,\;f_{\rho }\left(X,t\right)=0.$$

In this form, the technological constraints can be taken into account in optimization using Lagrange multipliers. In the notation of Equation (21) below, these Lagrange multipliers are $\mu_{\eta }$ and $\mu_{\rho }$.

The slack variables for labor, energy, and capital are $L_{\eta },E_{\eta }$ and $K_{\rho }$. They define the range in factor space within which the factors can vary independently at time *t*. Inserting them into Equation (14) yields the the explicit form of Equation (17) as

(18) $$f_{\eta }\left(X,t\right)\equiv \eta_{0}^{*}{\left(\frac{L+L_{\eta }}{K}\right)}^{\lambda }{\left(\frac{E+E_{\eta }}{K}\right)}^{\nu }-1=0,\;f_{\rho }\left(X,t\right)\equiv \frac{K+K_{\rho }}{K_{m}\left(Y\right)}-\rho_{T}\left(t\right)=0.$$

With respect to intertemporal optimization of overall welfare, Samuelson and Solow [27] state that “…society maximizes the (undiscounted) integral of all future untilities of consumption subject to the fact that the sum of current consumption and current capital formation is limited by what the current capital stock can produce”. A discount rate $\rho_{d}=0$ is also preferred by other leading economists like Ramsey [28] and Arrow [29] for ethical reasons. (In N. Stern’s famous (and by some economists, heavily criticized) Review Report on the Economics of Climate Change [30,31], future climate change damage is discounted with a rate of only 0.1; see also [8] (p. 243f)).

We follow the optimization procedure of Samuelson and Solow [27] with the following modifications: (a) as in [32], optimization is performed within finite time horizons; (b) there is not one variable production factor but three—$X_{1}$, $X_{2}$, and $X_{3}$; (c) they are constrained by Equation (18).

In addition to the technological constraints (18), there is the economic constraint on consumption; Equation (13) is the most general form. We consider the simple case, where the utility function *U* only depends on consumption *C*:

(19) U=U(C).

Consumption *C* is output Y(X,t) minus investment into new capital formation ${\dot{X}}_{1}\equiv dX_{1}/dt$:

(20) $$C\left(X,{\dot{X}}_{1}\right)=Y\left(X;t\right)-{\dot{X}}_{1}.$$

The additional economic constraint is that the total cost p·X of producing consumption *C* by means of the factors $(X_{1},X_{2},X_{3})\equiv X$ must not diverge. Rather, its magnitude $c_{f}\left(t\right)$ must be finite at all times *t*. (Each price per factor unit, $p_{i}$, is exogeneously given; the price of capital utilization $p_{1}$ is the sum of net interest, depreciation, and state influences):

(21) $$c_{f}\left(t\right)-\sum_{i=1}^{3}p_{i}\left(t\right)X_{i}\left(t\right)=0.$$

Therefore, besides $\mu_{\eta }$ and $\mu_{\rho }$, the variational formalism of intertemporal welfare optimization includes the Lagrange multiplier μ, which takes care of (21).

Thus, the optimization problem is maximize overall welfare:

(22) $$W\left[s\right]=\int_{t_{0}}^{t_{1}}dt\left\{U\left[C\left(X,{\dot{X}}_{1}\right)\right]+\mu \left[c_{f}\left(t\right)-p\cdot X\right]+\mu_{\eta }f_{\eta }\left(\vec{X},t\right)+\mu_{\rho }f_{\rho }\left(\vec{X},t\right)\right\}.$$

W[s] is a functional of the curve $\left[s\right]=\{t,X:\;X=X\left(t\right),\;t_{0}\leq t\leq t_{1}\}$. Consider another curve $\left[s,h\right]=\{t,X:\;X=X\left(t\right)+h\left(t\right),\;t_{0}\leq t\leq t_{1}\}$ close to [s], which goes through the same end points so that $h\left(t_{1}\right)=0=h\left(t_{0}\right)$. Its functional W[s,h] is obtained from W[s] by changing X to X+h and ${\dot{X}}_{1}$ to ${\dot{X}}_{1}+{\dot{h}}_{1}$ everywhere in the integrand of Equation (22). The necessary condition for the maximum of overall welfare is

(23) δW≡W[s,h]−W[s]=0.

The details of the variational calculus, performed in analogy to the derivation of the Lagrange equations from Hamilton’s principle of least action in classical mechanics, are in Appendix 2 of [8]. In the end, one finds that Equation (23) is satisfied if the output elasticities $\epsilon_{i}$ of the production factor $X_{i}$ satisfy the relation

(24) $$\epsilon_{i}\equiv \frac{X_{i}}{Y}\frac{\partial Y}{\partial X_{i}}=\frac{\mu X_{i}}{Y\frac{dU}{dC}}\left[p_{i}-\frac{\mu_{\eta }}{\mu }\frac{\partial f_{\eta }}{\partial X_{i}}-\frac{\mu_{\rho }}{\mu }\frac{\partial f_{\rho }}{\partial X_{i}}-\delta_{i,1}\frac{1}{\mu }\frac{d}{dt}\left(\frac{dU}{dC}\right)\right],$$

where i=1,2,3; the Kronecker delta $\delta_{i,1}$ is 1 for i=1 and 0 otherwise.

Just to bring out the importance of the technological constraints for the maximization of overall welfare, we simplify Equation (24) slightly by using a function of decreasing marginal utility of consumption $U\left(C\right)=C_{0}\ln\frac{C}{C_{0}}+U_{0}$. If one approximates it by its Taylor expansion up to first order in $\frac{C}{C_{0}}$, its time derivative vanishes. We continue with that.

The output elasticities (economic weights) of capital, labor, and energy satisfy the relation of constant returns to scale:

(25) $$\sum_{i=1}^{3}\epsilon_{i}=1.$$

Inserting $\epsilon_{i}$ from Equation (24) into Equation (25), one obtains the Lagrange multiplier that takes care of the constraint (21) as

(26) $$\mu =\frac{Y\frac{dU}{dC}}{\sum_{i=1}^{3}X_{i}\left[p_{i}-\frac{\mu_{\eta }}{\mu }\frac{\partial f_{\eta }}{\partial X_{i}}-\frac{\mu_{\rho }}{\mu }\frac{\partial f_{\rho }}{\partial X_{i}}\right]}.$$

With this μ, the equilibrium conditions (24) turn into

(27) $$\epsilon_{i}=\frac{X_{i}\left[p_{i}-\frac{\mu_{\eta }}{\mu }\frac{\partial f_{\eta }}{\partial X_{i}}-\frac{\mu_{\rho }}{\mu }\frac{\partial f_{\rho }}{\partial X_{i}}\right]}{\sum_{i=1}^{3}X_{i}\left[p_{i}-\frac{\mu_{\eta }}{\mu }\frac{\partial f_{\eta }}{\partial X_{i}}-\frac{\mu_{\rho }}{\mu }\frac{\partial f_{\rho }}{\partial X_{i}}\right]}.$$

The terms that are subtracted from the factor prices $p_{i}$ in Equation (27) make up the so-called *shadow prices*. Shadow prices of production factors that are subject to constraints in optimization translate these constraints into monetary terms. Their influence on the motion of the industrial sector of the Federal Republic of Germany in its cost mountain between 1960 and 1981 was computed and discussed in [12].

If there were no technological constraints, the Lagrange multipliers $\mu_{\eta }$ and $\mu_{\rho }$ would be zero and the output elasticities would be

(28) $$\epsilon_{i}=\frac{X_{i}p_{i}}{\sum_{i=1}^{3}X_{i}p_{i}},\;i=1,2,3.$$

This is the cost-share theorem.

As already mentioned in Section 3, according to this theorem, the economic weights of capital, $\epsilon_{1}\equiv \alpha$; labor, $\epsilon_{2}\equiv \beta$; and energy, $\epsilon_{3}\equiv \gamma$, are much larger for labor than for energy.

Since the 1990s, research on the anthropogenic greenhouse effect and climate change by top economists like Nordhaus and Schelling (Nobel price in economics 2005) has been conducted within the framework of mainstream economics and its cost-share theorem. They assumed that global warming only affects agriculture, which contributed less than 3% to the gross domestic product of the USA in 1992. (The 2009 share of US agriculture in GDP is a mere 0.6%.) The contribution of agriculture in other industrialized countries has been comparably low. Therefore, these scholars say that even a drastic decline in agricultural production should only result in small losses in welfare. Herman Daly commented on that in his paper “When smart people make dumb mistakes” [33].

Apparently, there is no memory of famines, which increase the value and the price of food dramatically and drastically enhance the contribution of agriculture to welfare.

The valuation of food and energy has been the same for economic sages: what costs little matters little. The constraints that result from the natural sciences and engineering are ignored. They should be taken into account, finally.

## 5. Outlook: Surmounting the Limits to Growth

So far, we have only considered industrial economies limited to the biosphere. Let us look beyond it.

After 1973, I had returned several times to Colombia for summer schools and conferences. On one occasion, in the early 1980s, I mentioned “The Limits to Growth” in a talk at the Universidad Nacional de Colombia in Bogotá. When I showed the slides of the break-down scenarios around the year 2030, a physics professor interrupted me and shouted “But you’ve forgotten outer space!”; I highly appreciated her interruption, in which she added that she is an optimistic Marxist, because industrial expansion into space is the only way to continue industrial growth and preserve the biosphere.

Gerard K. O’Neill’s 1974 publication *The Colonization of Space* [34], his research, and that of his collaborators at Princeton University and MIT on *The High Frontier* [35,36] stimulated hope in all who worried about limits to growth. I participated in a number of the “Princeton Conferences on Space Manufacturing Facilities”, and my wife Rita translated part of O’Neill’s book into German. To support the construction of space colonies that orbit around the Lagrange libration point L5, and solar power satellites, which would provide Earth with energy from the Sun, the *L5 Society* was formed. Its motto was “If you love the Earth, leave it”. More detailed reviews of these developments can be found in [8] (pp. 85–92) and [37].

With O’Neill’s premature death from leukemia and the waning interest in space on part of the USA, space colonization and solar power satellites were nearly forgotten. Now, they are being rediscovered [38,39], also by rapidly industrializing countries like China and India. Let us hope for peace, which abolishes arms races, and that humankind surmounts the limits to growth on Earth and opens up the treasures of Space.

## Data Availability

Data are contained within the article.
